# *Elizabethkingia miricola* bacteraemia in a haemodialysis patient

**DOI:** 10.1099/acmi.0.000098

**Published:** 2020-02-03

**Authors:** Julia C. Howard, Kevin Chen, Trevor Anderson, Simon C. Dalton

**Affiliations:** ^1^​ Microbiology Department, Canterbury Health Laboratories, Christchurch, New Zealand; ^2^​ Department of Infectious Diseases, Christchurch Hospital, New Zealand; ^†^​ Present address: Microbiology Department, Waikato Hospital, Hamilton, New Zealand; ^‡^​ Present address: Department of Medicine, Tauranga Hospital, Tauranga, New Zealand

**Keywords:** *Elizabethkingia*, bacteraemia, dialysis, line infection, antimicrobial

## Abstract

We report a case of catheter-associated *Elizabethkingia miricola *bacteraemia in a haemodialysis patient. The patient was a 73-year-old home haemodialysis patient who presented with a history of recurrent falls and fevers. Blood cultures grew Gram-negative bacilli identified by MALDI-TOF MS (matrix-assisted laser desorption/ionization time-of-flight mass spectrometry 6903 MSP Library) and 16S rRNA gene sequencing as *E. miricola. E. miricola* is an emerging human pathogen and is multidrug-resistant, making the choice of antimicrobial therapy challenging. There are only a small number of case reports of human infection worldwide and this is the second reported case of catheter-related bacteraemia. It has also been found in the hospital environment in South Korea and is pathogenic in black-spotted frogs.

## Case presentation

**Fig. 1.**
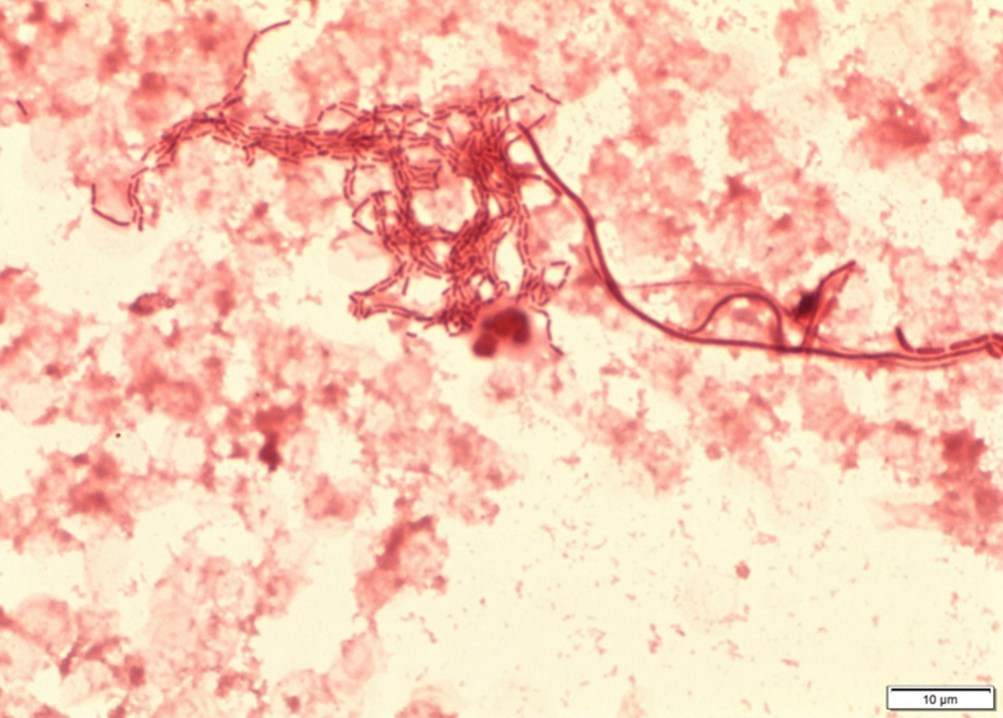
Gram stain showing pleomorphic Gram-negative bacilli. Bar, 10 µm.

A 73-year-old home haemodialysis patient presented to the Emergency Department at Christchurch Hospital with a history of recurrent falls and fevers during his home dialysis sessions over the last month. Blood tests showed a peripheral white cell count of 18.7×10^9^ (normal range 4–11×10^9^) and a C-reactive protein (CRP) of 183 mg l^−1^ (normal range 0–5 mg l^−1^). At initial presentation he was haemodynamically stable but in view of the clinical history he was admitted for observation. On day 1 of his admission he underwent haemodialysis in the dialysis unit and rapidly became clinically septic with tachycardia at 150 bpm, fever and rigors. His tunnelled line site did not look clinically infected. During the septic episode two sets of blood cultures were taken and he was started on ceftriaxone (1 g daily) and vancomycin (4 g loading dose followed by 2 g daily) empirically with a working diagnosis of line sepsis due to the timing of the septic episode. Ceftriaxone was started as he had a history of non-anaphylactoid penicillin allergy (mild rash). His CRP had risen to 222 mg l^−1^. On the second day of his admission his blood cultures from the arterial and venous lumens of his tunnelled dialysis line both flagged positive with an unusual pleomorphic Gram-negative bacillus (Fig. 1). Ciprofloxacin (250 mg twice daily) was added in to his therapeutic regimen as there was concern from the Gram stain morphology that the organism could be *Pseudomonas aeruginosa* and his line was removed. By day 3 he had improved clinically, his inflammatory markers were starting to decrease (peripheral white cell count 14.3×10^9^ and CRP 151 mg l^−1^) and he started to dialyse via his arterio-venous fistula, which had been created a few months earlier.

On the third day blood agar incubated in CO_2_ at 35–37 °C grew poorly growing oxidase-positive small translucent colonies. No other biochemical tests were performed and identification of the isolate was carried out using MALDI-TOF (matrix-assisted laser desorption/ionization time-of-flight) MS on the Bruker microflex LT (Bruker Daltonics, Bremen, Germany). Pure cultures of the isolate were inoculated onto the MALDI target plate using a wooden applicator, then 1 µl formic acid was applied and allowed to dry, and 1 µl HCCA matrix was applied and analysed with the6903 database. This identified the isolate as being *Elizabethkingia miricola* (score 2.38) with lower scores for *Elizabethkingia anopheles* (1.78) and *Elizabethkingia meningoseptica* (1.8). Routine Gram-negative disc susceptibility testing was undertaken. The organism was susceptible to ciprofloxacin and piperacillin-tazobactam and there was growth up to the trimethoprim/sulfamethoxazole disc with no visible zone. E-tests were performed as per the manufacturer’s instructions. The isolate underwent our laboratory's routine Gram-negative susceptibility testing using the BD Phoenix^TM^ automated microbiology system. No EUCAST clinical breakpoints are available for *Elizabethkingia* species but using EUCAST non-species-specific PK/PD breakpoints showed it was susceptible to ciprofloxacin (MIC=0.064 mg l^−1^) and levofloxacin (MIC=0.38 mg l^−1^), but resistant to ampicillin, amoxicillin-clavulanate, cefuroxime, ceftriaxone, meropenem, colistin and gentamicin. No other micro-organisms were cultured from the patient. Given that this is an unusual human pathogen, we performed 16S rRNA gene sequence analysis in our laboratory (Canterbury Health laboratories, Christchurch). For DNA extraction, a pure culture isolate was lysed by mechanical disruption using 0.5 mm ceramic beads and DNA was purified on the EasyMag platform using the specific A 1.0.2 protocol. The 5′ region of the 16S rRNA gene was amplified using PCR primers and thermal cycling conditions as described by Wilbrink *et al*. [1]. The amplicon was purified and sequence reaction was performed using BDT v3.1 chemistry and analysed on an ABI3130xl genetic analyser (Applied Biosystems) as recommended by the manufacturer. The 16S rRNA gene sequence was aligned with known sequences in GenBank using blastn and analysis using the Quick BioInformatic Phylogeny of Prokaryotes – QBPP Database (https://umr5558-bibiserv.univ-lyon1.fr/lebibi/lebibi.cgi) [2]. The sequence alignment in GenBank was a 100 % (497/497 nt) match to *E. miricola* (accession number CP023746.1) isolated from a blood specimen from a hospital in Taiwan [3] and the phylogenetic tree showed clustering of *E. miricola*, *E. occulta* and *E. bruuniana*. The sequence has been submitted to GenBank (accession number MN630847). The combination of the MALDI-TOF and 16S rRNA gene sequencing confirmed identification as *E. miricola* [4]. A peripheral blood culture taken 48 h after starting antibiotics showed no growth at 5 days of incubation. A culture of the line tip also grew Gram-negative bacilli, which were identified by MALDI-TOF MS as *E. miricola*. The patient's antibiotic regimen was rationalized to oral ciprofloxacin 500 mg (the dose had been increased to 500 mg) twice daily for a 10-day course. He was discharged home on day 17 to complete a total course of 3 weeks of antibiotics and made a good recovery. At discharge his peripheral white cell count was 13.4×10^9^ and his CRP was 125 mg l^−1^. There were no other cases identified in the hospital and we did not carry out any environmental testing.

## Discussion

E. miricola is a multidrug-resistant emerging human Gram-negative pathogen [5]. It was formerly known as *Chryseobacterium miricola* and was first isolated in 2003 from condensation water on the space station Mir [6]. It is a non-fermenter and is oxidase- and indole-positive [7]. It has been reported as causing infection in a stem-cell transplant recipient with lymphoma who had bacteraemia and ventilator-associated pneumonia in 2008 in the USA [8], community-onset urinary tract infection (UTI) in a patient with pre-existing hydronephrosis and vesicoureteric reflux in India [9], nosocomial pneumonia in a postoperative spinal patient [5] and UTI in a co-morbid child in Switzerland [10], knee septic arthritis in a patient with recurrent erysipelas in Denmark [4], catheter-related bacteraemia in a diabetic patient with cardiomyopathy in Hong Kong [11], bacteraemia in a patient with alcoholic pancreatitis in Italy [12], and pulmonary abscesses in a septic patient in France [13]. There is also a case report of the organism acting as an opportunistic pathogen causing oral superinfection in a patient with common variable immunodeficiency in Poland [14], and the first reported case of pulmonary exacerbation in a cystic fibrosis (CF) patient was recently reported from the UK [15]. In addition, it has been isolated from environmental specimens taken from ward washbasins in a hospital in South Korea [16].

Until recently there have been three recognized species of *Elizabethkingia *(*E. meningoseptica*, *E. miricola* and *E. anopheles*); however a study in the UK which analysed 44 *Elizabethkingia* species isolates from clinical specimens (sputa, bronchoalveolar lavage, cough swabs and tracheal aspirates) in CF patients using *rpoB* gene sequencing has shown that an *E. miricola* cluster exists and includes *E. miricola* as well as the proposed novel species *E. occulta* sp. nov., *E. ursingii* sp. nov. and *E. bruunniana* sp. nov. [17]. These species were also proposed by the Centers for Disease Control in a taxonomic review of the genus *Elizabthkingia* in 2018 [18]. There is also evidence of its pathogenic potential in animals as it has also been reported to cause epidemic meningitis-like disease in black-spotted frogs farmed for human consumption in China in 2016 [19]. Like *E. meningoseptica*, which is more commonly found as a cause of nosocomial infection in patients who have received prior courses of antibiotic therapy, it is multidrug-resistant [5], making the choice of optimal therapy difficult. Some strains may contain one or more metallo-β-lactamase genes [5, 10], as is likely in our case given the susceptibility results. Treatment that has been successful in the medical literature includes tigecycline and levofloxacin combination therapy [8], piperacillin-tazobactam and ciprofloxacin [13], piperacillin-tazobactam [4, 9], imipenem/cilastin and ciprofloxacin followed by piperacillin-tazobactam and ciprofloxacin [12], levofloxacin [11, 14], and ciprofloxacin [4, 15]. In our case ciprofloxacin was the only clinically available option as levofloxacin is not available in New Zealand, the organism was resistant to trimethoprim/sulfamethoxazole and the patient had a penicillin allergy (rash). Options for therapy may include fluoroquinolones, trimethoprim/sulfamethoxazole and piperacillin-tazobactam depending on the organism’s susceptibility profile and patient medication contraindications.

In conclusion, we describe a catheter-associated bacteraemia caused by the emerging pathogen *E. miricola* in a haemodialysis patient, and which was successfully treated with ciprofloxacin combined with dialysis line removal.
